# A Purified Recombinant Lipopeptide as Adjuvant for Cancer Immunotherapy

**DOI:** 10.1155/2014/349783

**Published:** 2014-03-11

**Authors:** Ying-Chyi Song, Hsueh-Hung Liu, I-Hua Chen, Hsin-Wei Chen, Pele Chong, Chih-Hsiang Leng, Shih-Jen Liu

**Affiliations:** ^1^National Institute of Infectious Diseases and Vaccinology, National Health Research Institutes, Zhunan, Miaoli 35053, Taiwan; ^2^Graduate Institute of Immunology, China Medical University, Taichung 40447, Taiwan

## Abstract

Synthetic lipopeptides have been widely used as vaccine adjuvants to enhance immune responses. The present study demonstrated that the tryptic N-terminal fragment of the lipoprotein rlipo-D1E3 (lipo-Nter) induces superior antitumor effects compared to a synthetic lipopeptide. The lipo-Nter was purified and formulated with protein or peptide vaccines to determine if lipo-Nter could be used as a novel adjuvant and could induce antitumor immunity in a cervical cancer model. Purified lipo-Nter activated the maturation of bone marrow-derived dendritic cells (BM-DCs), leading to the secretion of TNF-**α** through TLR2/6 but not TLR1/2. A recombinant mutant HPV16 E7 (rE7m) protein was mixed with lipo-Nter to immunize the mice; the anti-E7 antibody titers were increased, and the T helper cells were skewed toward the Th1 fate (increased IL-2 and decreased IL-5 secretion). Single-dose injection of rE7m and lipo-Nter inhibited tumor growth, but the injection of rE7m alone did not. Accordingly, lipo-Nter also enhanced the antitumor immunity of the E7-derived peptide but not the synthetic lipopeptide (Pam3CSK4). We demonstrated that the lipo-Nter of a bacterial-derived recombinant lipoprotein is a novel adjuvant that could be used for the development of a new generation of vaccines.

## 1. Introduction

The discovery of Toll-like receptors (TLR) and their ligands has enabled the development of novel adjuvants that can enhance the adaptive immune response toward specific antigens. A number of novel TLR agonists have entered the clinical arena as vaccine adjuvants and are even used as immune modulators in monotherapy [1]. TLR ligands act as potential adjuvants that control DC maturation and influence the magnitude of T cell responses [2–4]. Certain lipoproteins of bacteria are recognized by TLR2 or TLR4 and can induce the activation of antigen-presenting cells (APCs) [5–9]. Importantly, di- or triacylated S-(2,3-dihydroxypropyl) cysteines in the N-terminal lipopeptides are essential for TLR2 activation [10, 11]. There is evidence that TLR2 can form heterodimers with TLR1 or TLR6, further defining their ligand specificity. The diacylated or triacylated lipopeptide is recognized by TLR2/TLR6 and TLR2/TLR1 heterodimers, respectively [5, 12, 13]. However, studies have also suggested that both the fatty acid residues and the N-terminal amino acid domain of the lipopeptide contribute to the specificity of its recognition by TLR1/TLR2 or TLR2/TLR6 heterodimers [11]. In addition, heterodimerization of TLR2 with TLR1 or TLR6 does not lead to the activation of different signaling pathways but rather expands the ligand spectrum [14]. We previously demonstrated that a recombinant lipoprotein containing unsaturated fatty acids activates TLR2 signaling through a TLR1- and TLR6-independent pathway [15]. However, the relationship between the number of fatty acids in the lipopeptide and the involvement of the TLR2 coreceptor remains unclear.

Synthetic lipopeptides derived from bacterial lipoproteins are effective adjuvants for vaccination that elicit both Th1 and Th2 cytokines depending on the model antigen used in the immunizations. The diacylated lipopeptide FSL-1 possesses TLR2-mediated adjuvant activity to induce T helper 2 (Th2) type responses in vivo [16]. However, the synthetic triacylated lipopeptide corresponding to the N-terminal sequences of* B. burgdorferi *outer surface lipoproteins can induce Th1 phenotype development [17]. Furthermore, the lipopeptide N-Palmitoyl-S-[2,3-bis(palmitoyloxy)-(2RS)-propyl]-CSKKKK, Pam3CSK4, a synthetic analog of bacterial and mycoplasmal lipoproteins that recognizes the TLR1/2 complex, can enhance functional memory CD8^+^ T cells [18, 19] and facilitate antigen-driven CD4^+^ T cell differentiation [20]. A synthetic derivative of the* mycoplasma* macrophage-activating lipopeptide-2, BPPcysMPEG, is a potent adjuvant for cross-priming against cellular antigens [21]. These studies indicate that synthetic lipopeptides are capable of acting as adjuvants for vaccine development.

Recently, we developed a novel technology for expressing high levels of recombinant rlipo-D1E3 (lipidated dengue virus envelope protein domain 3) with intrinsic adjuvant properties in* Escherichia coli* C43 (DE3) [22]. We demonstrated that the lipid moiety of this* E. coli*-derived recombinant lipoprotein contains unsaturated fatty acids with different chain lengths [23] and that this recombinant lipoprotein can activate NF-*κ*B through the TLR2 signaling pathway, resulting in a different cytokine profile from that induced by the synthetic lipopeptide (Pam3CSK4) in BM-DCs [15].

In this study, we purified the tryptic N-terminal fragment of rlipo-D1E3 (lipo-Nter) and determined if lipo-Nter could be used as a novel adjuvant in a peptide or protein vaccine. The ability of antigens formulated with lipo-Nter to activate BM-DCs, to elicit B and T cell immune responses, and to increase the presentation levels of the T cell epitope were investigated. Different TLR knockout mice were used to determine the coreceptor usage when stimulating the cells with Pam3CSK4 or lipo-Nter. Furthermore, we examined the antitumor effects of Pam3CSK4 or lipo-Nter formulated with a peptide vaccine in tumor-bearing mice. These results not only provide information on the use of lipo-Nter as a novel adjuvant but also demonstrate that the lipid structure of a lipopeptide can affect the coreceptor it binds to and can induce different levels of antitumor effects.

## 2. Materials and Methods 

### 2.1. Cell Lines and Medium

TC-1, a mouse epithelial cell line transformed with the oncogenes Ras, HPV16 E6, and E7, was a kind gift from Dr. T-C. Wu (Johns Hopkins University, USA). The TC-1 cells were cultured in DMEM (GIBCO-BRL, Grand Island, NY) supplemented with 10% heat-inactivated fetal bovine serum (HyClone, Logan, Utah), penicillin (100 U/mL), and streptomycin (100 *μ*g/mL) (GIBCO-BRL, NY, USA).

### 2.2. Preparation of Lipidated N-Terminal Fragments (Lipo-Nter) from Rlipo-D1E3

The preparation and purification of recombinant rlipoD1E3 have been described previously [22]. A total of 100 mg of purified rlipo-D1E3 was digested with trypsin at a ratio of 50 : 1 at room temperature for 4 h. The reaction was then stopped by adding 100% formic acid at a ratio of 100 : 3, and the mixture was loaded onto 7.2 g C18 silica gel (Fluka, Buchs, Switzerland) that had been suspended in 200 mL 100% acetonitrile (ACN) and preequilibrated with 80 mL 0.1% trifluoroacetic acid (TFA). A total of 100 mg digested rlipo-D1E3 was loaded into the C18 column. The column was washed with 200 mL 0.1% TFA, followed by 400 mL 70% ACN/0.1% TFA. The final washing was performed with 120 mL 100% ACN. The lipo-Nter was then eluted with 40 mL isopropanol. The yield of lipo-Nter after elution with isopropanol was 18%.

### 2.3. Peptide Synthesis

The H-2D^b^-restricted CTL epitope (amino acids 49–57, RAHYNIVTF) (RAH) derived from the HPV16 E7 protein was purchased from GL Biochem (Shanghai, China). The lipopeptide Pam3CSK4 (chemical name: N-Palmitoyl-S-[2,3-bis(palmitoyloxy)-(2RS)-propyl]-[R]-cysteinyl-[S]-seryl-[S]-lysyl-[S]-lysyl-[S]-lysyl-[S]-lysine), a synthetic analog of bacterial and mycoplasmal lipoproteins, was purchased from GeneDireX (Nevada, USA). The purity of all peptides was >85%. All peptides were dissolved in DMSO or PBS at a concentration of 10 mg/mL and stored at −80°C until use.

### 2.4. Animals

Female C57BL/6 mice, 6–12 weeks of age, were obtained from the National Laboratory Animal Breeding and Research Center (Taipei, Taiwan). TLR1^−/−^(TLR1 KO), TLR2^−/−^ (TLR2 KO), and TLR6^−/−^ (TLR6 KO) mice were purchased from Oriental BioService (Osaka, Japan). All animals were housed at the Animal Center of the National Health Research Institutes (NHRI) and maintained in accordance with the institutional animal care protocol. All animal studies were approved by the animal committee of NHRI.

### 2.5. BM-DC Isolation and Maturation

Bone marrow cells from C57BL/6, TLR1^−/−^, TLR2^−/−^, or TLR6^−/−^ mice were cultured at a density of 2 × 10^5^ cells/mL in Petri dishes containing 10 mL complete RPMI-1640 medium (Gibco, NY, USA) with 20 ng/mL recombinant mouse granulocyte-macrophage colony-stimulating factor (GM-CSF; Peprotech Inc., New Jersey, USA). Complete RPMI-1640 medium consisted of RPMI-1640 supplemented with 10% (v/v) heat-inactivated fetal calf serum, 25 mM HEPES (Biological industries, Beit Haemek, Israel), 100 units/mL penicillin, 100 *μ*g/mL streptomycin sulfate, and 50 *μ*M *β*-mercaptoethanol (Sigma, MO, USA). On day 3, another 10 mL of complete RPMI medium containing 20 ng/mL GM-CSF was added. On day 6, the cells were collected from each dish, washed, and counted.

To investigate the effect of lipo-Nter and Pam3CSK4 on the functional maturation of DCs, 1 × 10^6^ DCs/mL were plated in complete RPMI-1640 medium. Lipo-Nter (10 *μ*g/mL) was then added, and the cells were further incubated for 24 h. As a positive control, the cells were incubated with 0.1 *μ*g/mL lipopolysaccharide (LPS). After incubation, the supernatants of the cultured cells were isolated and assayed for TNF-*α* using a DuoSet ELISA kit (R&D Systems, MN, USA) according to the manufacturer's protocol.

### 2.6. Antibody Titer

C57BL/6 mice were subcutaneously administered 30 *μ*g rE7m mixed with/without 30 *μ*g/100 *μ*L lipo-Nter twice at a 2-week interval. Serum samples were collected at week 4, and anti-rE7m antibody titers were determined by a sandwich ELISA. In brief, 50 *μ*L (10 *μ*g/mL) of purified rE7m was coated in 96-well microtiter plates with 0.1 M carbonate buffer (pH 9.6) by overnight incubation at 4°C. The coated plates were washed twice with PBST and then blocked with 5% nonfat milk in PBS at room temperature for 2 h. The diluted sera from immunized animals were applied to the wells at room temperature for 2 h. After incubation with HRP-conjugated goat anti-mouse IgG (Sigma, St. Louis, MO, USA), the assay was developed with 3,3′,5,5′-tetramethylbenzidine (TMB), and the reaction was stopped by adding 100 *μ*L 1 M H_2_SO_4_ to each well. The plates were read at 450 nm using an ELISA plate reader (Molecular Devices, Sunnyvale, CA, USA). For antibody isotype analysis, biotin-conjugated rat anti-mouse IgG1 and IgG2b (BD Pharmingen, San Diego, CA, USA) were added to the wells for sera binding; then, HRP-conjugated streptavidin was added, followed by development with the TMB substrate. The antibody titer was defined as the reciprocal of the highest dilution that produced an OD450 value 2-fold higher than that of the preimmune sera.

### 2.7. Cytokine ELISA

Wild-type C57BL/6 mice were immunized twice at a 2-week interval with PBS or rE7m (30 *μ*g) mixed with/without lipo-Nter (30 *μ*g/100 *μ*L). On day 7 after the second immunization, splenocytes (1 × 10^6^/mL) were stimulated with rE7m (100 nM) for 3, 4, or 5 days. The supernatant of the cultured cells was isolated and assayed for IL-2, IFN-*γ*, and IL-5 using a DuoSet ELISA kit (R&D Systems, MN, USA) according to the manufacturer's protocol.

### 2.8. ELISPOT Assay

IFN-*γ*-secreting cells were analyzed using an IFN-*γ* ELISPOT assay as previously described [15]. Briefly, splenocytes (5 × 10^5^/well) were added to anti-IFN-*γ*-coated plates and cultured in the presence of 10 *μ*g/mL of the indicated peptides in a final volume of 200 mL RPMI-10. After incubation, the cells were removed by washing the plates with 0.05% (w/v) Tween 20 in PBS. A 50 mL aliquot containing 10 *μ*g/mL of biotinylated anti-IFN-*γ* antibody (clone R46A2, eBioscience San Diego, CA) was added to each well, and the samples were incubated for 2 h. The spots were developed using 3-amine-9-ethyl carbazole (Sigma, St. Louis, MO) and counted using an ELISPOT reader (Cellular Technology Ltd., Shaker Heights, OH).

### 2.9. Tumor Model

C57BL/6 mice were injected with 2 × 10^5^ TC-1 cells in the abdominal region. After 7 days, TC-1 tumor-bearing mice (6 animals/group) received a single injection of 100 *μ*L PBS, rE7m (30 *μ*g) mixed with/without lipo-Nter (30 *μ*g/100 *μ*L), or RAH (10 *μ*g) mixed with lipo-Nter (10 *μ*g/100 *μ*L) or Pam3CSK4 (10 *μ*g/100 *μ*L). The tumor diameters were measured in two orthogonal dimensions using a caliper two or three times per week. The tumor volumes were calculated from the measurements according to the following formula: (length × width^2^)/2. The tumor diameters are shown (cm^3^).

### 2.10. CD8^+^ T Cell Proliferation

Mice were vaccinated by two subcutaneous (s.c.) injections of the H-2D^b^-restricted E7-derived peptide (RAH: RAHYNIVTF) (30 *μ*g/100 *μ*L) mixed with 30 *μ*g of a pan-DR T-helper epitope (PADRE: AKFVAAWTLKAAA) in 100 *μ*L incomplete Freund's adjuvant (IFA) 1 week apart. Splenocytes were harvested, and CD8^+^ T cells were isolated by magnetic purification using a Dynal mouse CD8 negative isolation kit (Oslo, Norway) according to the manufacturer's instructions. The purity of the CD8^+^ T cells was then determined by flow cytometry using a FITC-conjugated anti-CD8 mAb. After magnetic purification, the cell preparation consisted of >85% CD8^+^ T cells (data not shown). The purified CD8^+^ T cells (2 × 10^5^) were cocultured with/without DCs at 2 × 10^4^ per well in 96-well plates and stimulated with PBS or 1 *μ*M RAH mixed with PBS or 200 nM lipo-Nter. Subsequently, 1 *μ*Ci of [^3^H]thymidine (specific activity 83 Ci/mmol; PerkinElmer, MA, USA) was added to each well for the last 18 h of a 72 h culture. The cells were harvested, and [^3^H]thymidine uptake was determined using a TopCount NXT microplate scintillation counter.

## 3. Results

### 3.1. Characterization of Lipo-Nter

**Figure 1:**
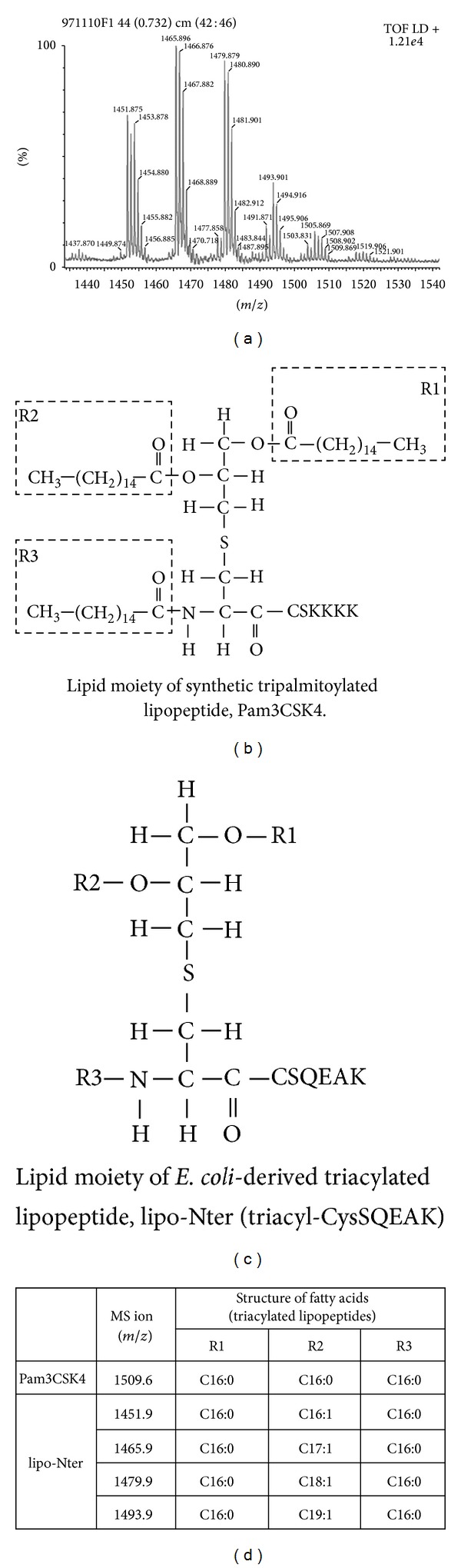
The lipid structures of Pam3CSK4 and Triacyl-CysSQEAK (lipo-Nter). (a) Identification of purified lipo-Nter by mass spectrometry. Lipo-Nter was obtained after trypsinizing rlipo-D1E3 and was analyzed on a Waters MALDI micro MX mass spectrometer. The MALDI-TOF MS spectra revealed the existence of four peaks with* m/z* values of 1451.9, 1465.9, 1479.9, and 1493.9. (b) Pam3CSK4 contains an N-acyl-S-diacylglyceryl cysteine moiety, and the fatty acids in all of the groups are 16-carbon saturated fatty acids. (c) The lipid structure of lipo-Nter is also an N-acyl-S-diacylglyceryl cysteine structure. The R2 group of lipo-Nter contains unsaturated fatty acids with different chain lengths. (d) The masses of Pam3CSK4 and lipo-Nter were determined. We previously [23] identified the masses of the corresponding lipid structures.

To obtain lipidated N-terminal fragments (lipo-Nter) of rlipo-D1E3 to evaluate its adjuvanticity, rlipo-D1E3 was digested with trypsin and purified from the digestion mixture by chromatography on C18 silica resin. The purified lipo-Nter was then analyzed by mass spectroscopy, which revealed the existence of four peaks with* m/z* values of 1451.9, 1465.9, 1479.9, and 1493.9 (Figure 1(a)). The lipid modifications of rlipo-D1E3 have been previously identified and characterized by mass spectrometry [23]. The lipid structure of Pam3CSK4 is tripalmitoylated (Figure 1(b)), in contrast to the lipid structure of lipo-Nter at the R2 position, which contains an unsaturated fatty acid with a different chain length. In addition, the amino acid moiety of lipo-Nter is CSQEAK (Figure 1(c)). This study aimed to determine whether lipo-Nter, with its different fatty acid structure, could also be used as an adjuvant.

### 3.2. Lipo-Nter Induces BM-DC Activation through TLR2 and TLR6

**Figure 2:**
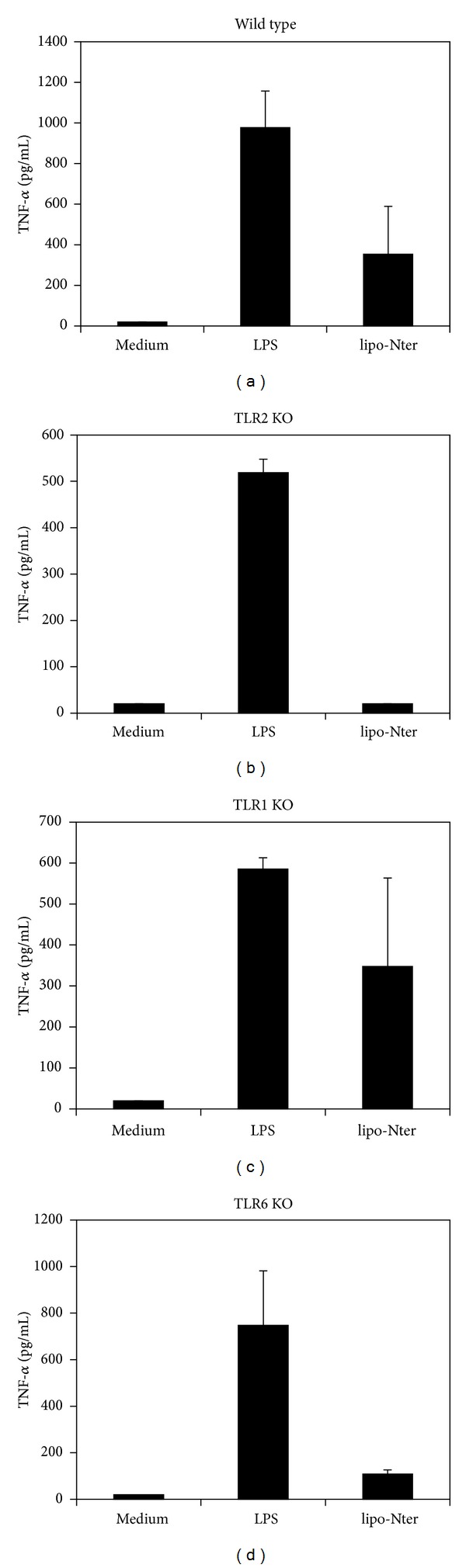
The responses of BM-DCs after stimulation with lipo-Nter. BM-DCs were generated from wild-type (WT), TLR1^−/−^ (TLR1 KO), TLR2^−/−^ (TLR2 KO), or TLR6^−/−^ (TLR6 KO) mice. Different types of BM-DCs were stimulated with/without LPS (0.1 *μ*g/mL) or lipo-Nter (100 nM) for 24 h. The levels of TNF- in the culture supernatant were measured by ELISA. LPS (TLR4 agonist) was used as positive control. The results are expressed as the means + S.D. of the amount of cytokine.

Di- or triacylated lipopeptides are recognized by TLR2/TLR6 or TLR2/TLR1 heterodimers, respectively [5, 12]. To determine if TLR1, TLR2, or TLR6 is recognized by lipo-Nter, we used BM-DCs derived from wild-type, TLR1^−/−^, TLR2^−/−^, or TLR6^−/−^ mice as a model to study the activation of APCs. As shown in Figure 2, lipo-Nter can stimulate the production of TNF-*α* by the BM-DCs of wild-type mice. The stimulating effect of lipo-Nter was absent in the TLR2^−/−^ and TLR6^−/−^ mice but was present in the TLR1^−/−^ and wild-type mice (Figure 2). These results indicate that the TLR2 and TLR6 proteins are necessary for lipo-Nter-induced cytokine production by BM-DCs.

### 3.3. Lipo-Nter Triggers Changes in the Th1/Th2 Balance

**Figure 3:**
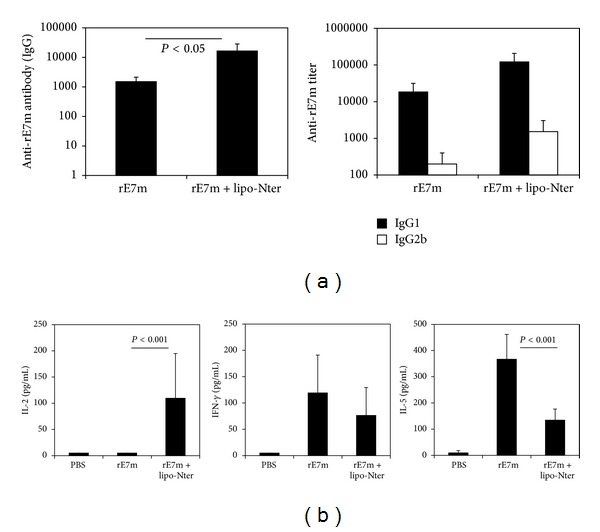
IgG isotype and T cell cytokine profile induced in mice following immunization with rE7m mixed with lipo-Nter. Wild-type C57BL/6 mice were subcutaneously administered rE7m (30 *μ*g) mixed with/without lipo-Nter (30 *μ*g/100 *μ*L) or PBS twice at a 2-week interval. (a) After 2 weeks, serum samples were collected, and anti-rE7m antibody titers were determined by sandwich ELISA. (b) On day 7 after the second immunization, splenocytes from immunized mice were stimulated with rE7m (100 nM), and the levels of IL-2, IFN-*γ*, and IL-5 in the culture supernatant were measured by ELISA. The data are expressed as the means + S.D. of the samples.

To investigate the adjuvant properties of lipo-Nter in vivo, a recombinant mutant E7 protein of HPV16 (rE7m) was used as a model [24]. Mice were immunized with either lipo-Nter/rE7m or rE7m alone, and the magnitudes of the antigen-specific antibody responses in the mice were analyzed. The antibody titers elicited by lipo-Nter/rE7m were 10-fold higher than those elicited by rE7m (Figure 3(a)). IgG isotype analysis revealed that although IgG1 (a marker of the Th2 response) and IgG2b (a marker of the Th1 response) were induced by both lipo-Nter/rE7m and rE7m, immunization with lipo-Nter/rE7m resulted in a lower serum IgG : IgG2b ratio (IgG1/IgG2b = 80) compared to rE7m immunization (IgG1/IgG2b = 92). This result suggests that lipo-Nter/rE7m immunization induces a polarized Th1 response (Figure 3(a)). Furthermore, we evaluated the adjuvant effects of lipo-Nter on antigen-specific T cell responses. Splenocytes from mice immunized with lipo-Nter/rE7m or rE7m were restimulated with rE7m, and supernatants were collected to analyze cytokine (IL-2, IFN-*γ*, and IL-5) production levels. The levels of IL-2 in the lipo-Nter/rE7m immunization group were 100-fold higher than those of the rE7m immunization group (Figure 3(b)). There was no significant difference in the secretion of IFN-*γ* (Th1 cytokine) between immunization with rE7m and lipo-Nter/rE7m. However, immunization with lipo-Nter/rE7m induced a 3-fold lower level of IL-5 (Th2 cytokine) than that induced by rE7m immunization (Figure 3(b)). These results demonstrate that immunization with lipo-Nter/rE7m attenuates the Th2 response of the recombinant protein rE7m. Thus, lipo-Nter potentially triggers a bias toward Th1 responses when added to protein vaccines.

### 3.4. Lipo-Nter Enhances Antigen-Specific T cell Responses and the Antitumor Effects of Recombinant Protein

**Figure 4:**
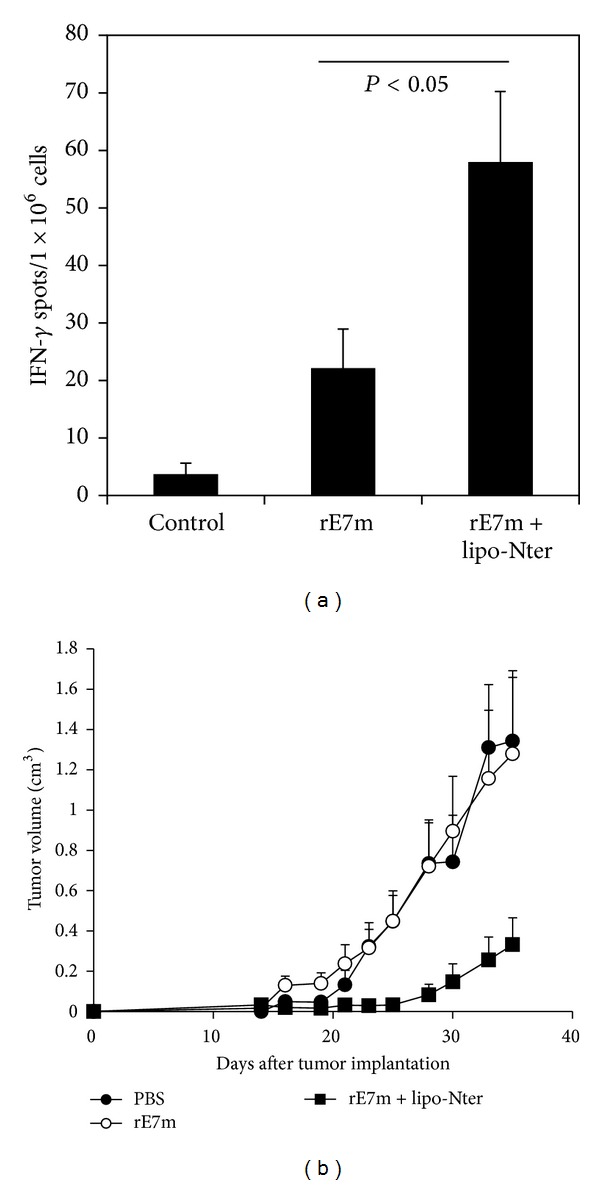
CD8^+^ T cell response and antitumor effect elicited by lipo-Nter in a protein vaccine. (a) C57BL/6 mice were immunized twice by subcutaneous injection with PBS or rE7m (30 *μ*g) mixed with/without lipo-Nter (30 *μ*g/100 *μ*L) at a 2-week interval. On day 7 after the second immunization, the mice were sacrificed, and splenocytes (2 × 10^5^ cells/well) were stimulated with or without 10 *μ*g/mL RAHYNIVTF (RAH) peptide for 48 h in an anti-IFN-*γ*-coated 96-well ELISPOT plate. The IFN-*γ*-secreting spots were measured using an ELISPOT reader. (b) 2 × 10^5^ TC-1 cells were injected into the abdominal region of the mice. After 7 days, TC-1 tumor-bearing mice (6 animals/group) received a single injection with PBS or rE7m (30 *μ*g) mixed with or without lipo-Nter (30 *μ*g/100 *μ*L). The tumor diameters are shown (cm^3^). The data are expressed as the means + SD of 6 animals per group.

TLR ligands serve as adjuvants to provide additional costimulatory signals and to induce cytokine production for T cell priming [2–4]. Based on our results, we investigated the induction of cytotoxic T lymphocyte (CTL) responses in the presence of lipo-Nter. C57BL/6 mice were subcutaneously immunized with lipo-Nter/rE7m, rE7m, or a PBS control. The splenocytes of the immunized mice were stimulated with a CTL epitope of E7 (RAHYNIVTF (RAH), E7_49–57_), and the number of RAH-specific IFN-*γ*-secreting cells was determined using the ELISPOT assay. Immunization with lipo-Nter/rE7m induced a higher number of RAH-specific IFN-*γ*-secreting cells than that induced by rE7m immunization (Figure 4(a)). Furthermore, we studied the efficiency of lipo-Nter for inducing an antitumor response. TC-1 tumor-bearing mice were immunized once with rE7m mixed with PBS or lipo-Nter. As shown in Figure 4(b), immunization with lipo-Nter/rE7m significantly delayed tumor growth. These data demonstrate that the lipo-Nter adjuvant provides a robust specific T cell response and antitumor activity in protein vaccines.

### 3.5. Lipo-Nter Elicits Specific T Cell Responses to a Peptide Vaccine

**Figure 5:**
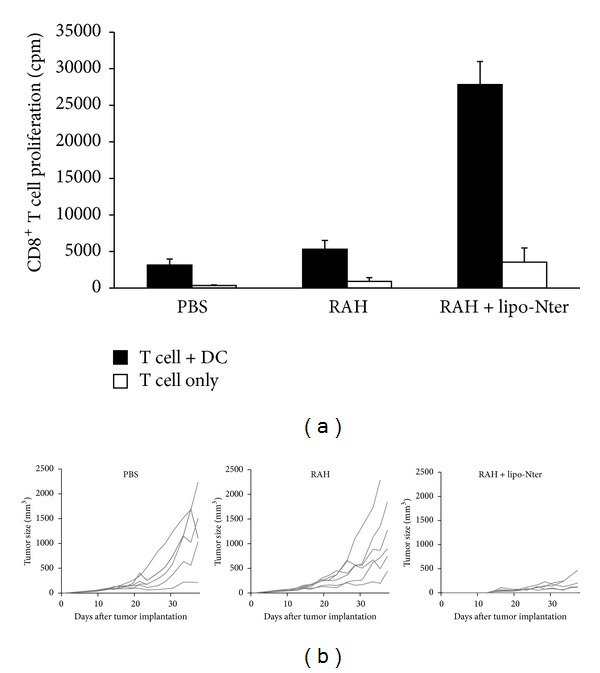
CD8^+^ T cell response and antitumor effect elicited by lipo-Nter in a peptide vaccine. (a) Mice were subcutaneously administered twice at a 1-week interval with RAH and PADRE (30 *μ*g) mixed with IFA. CD8^+^ T cells were purified from the spleens of the immunized mice. Purified CD8^+^ T cells were cocultured with/without BM-DCs and stimulated with PBS or 1 *μ*M RAH mixed with/without 200 nM lipo-Nter for 72 h. Proliferation of T lymphocytes was measured by uptake of  [^3^H]thymidine. (b) TC-1 tumor-bearing mice (6 animals/group) received a single injection of PBS or 10 *μ*g of RAH mixed with/without lipo-Nter (10 *μ*g/100 *μ*L). The tumor diameters are shown (cm^3^).

To further demonstrate the efficiency of lipo-Nter for enhancing the specific T cell response to a synthetic peptide in addition to a recombinant protein, C57BL/6 mice were subcutaneously immunized with the HPV 16 E7-derived peptide (RAH). CD8^+^ T cells were purified from the spleens of RAH-immunized mice and cocultured with DCs that were pulsed with lipo-Nter/RAH, RAH, or a PBS control. The proliferation of CD8^+^ T cells induced by the stimulation with lipo-Nter/RAH was 5-fold higher than that induced by RAH stimulation alone (Figure 5(a)). To confirm the enhancement of CD8^+^ T cells by lipo-Nter in vivo, tumor-bearing mice received a single injection of lipo-Nter/RAH, RAH, or a PBS control subcutaneously. The result was similar to that obtained for the recombinant protein vaccine; the RAH peptide mixed with lipo-Nter significantly inhibited tumor growth (Figure 5(b)). These data demonstrate that the use of lipo-Nter as an adjuvant enhanced the antigen-specific T cell response and antitumor activity of both protein and peptide vaccines.

### 3.6. A Synthetic Lipopeptide as an Adjuvant Cannot Efficiently Induce Anti-Tumor Effects

**Figure 6:**
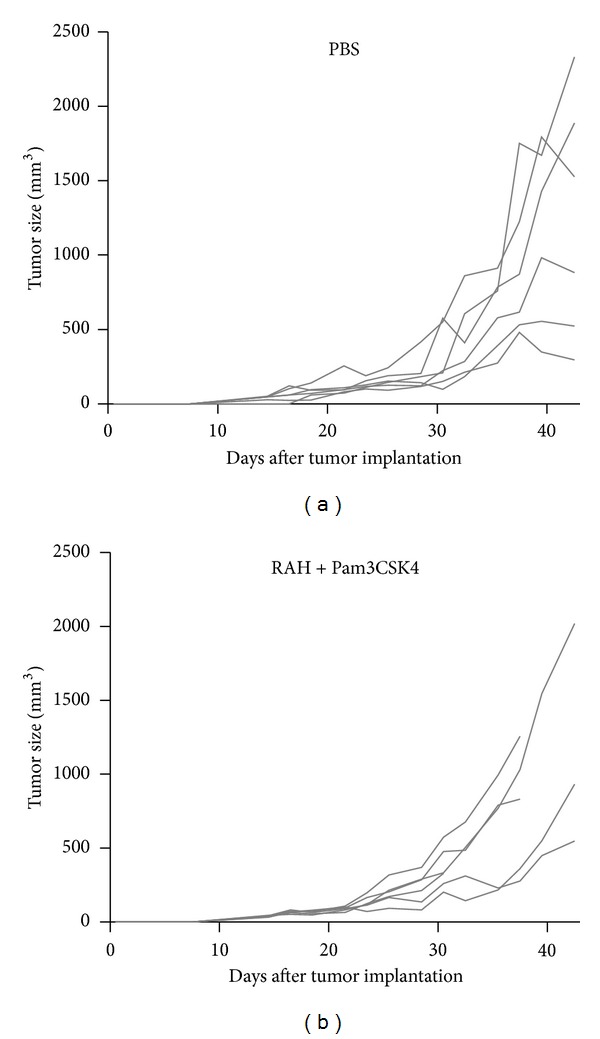
Anti-tumor effects of Pam3CSK4. TC-1 tumor-bearing mice (5 or 6 animals/group) received a single injection of PBS or 10 *μ*g of RAH mixed with Pam3CSK4 (10 *μ*g/100 *μ*L). The tumor diameters are shown (cm^3^).

The lipid moiety structure of lipo-Nter is a triacyl lipid that contains two saturated fatty acids and one unsaturated fatty acid (Figure 1). The tri-palmitoylated lipopeptide (Pam3CSK4) was previously shown to enhance the effector functions of CD8^+^ T cells [25]. Therefore, we assessed whether the lipo-Nter-induced anti-tumor immunity of the peptide vaccine is comparable to that induced by Pam3CSK4. We previously demonstrated that Pam3CSK4 activates BM-DCs through either TLR1/TLR2 or TLR6/TLR2 [15]. In contrast to Pam3CSK4, TLR2/TLR6 is necessary for lipo-Nter (Figure 2). We further evaluated the anti-tumor effects of Pam3CSK4 as an adjuvant for a peptide vaccine. TC-1 tumor-bearing mice were injected once with Pam3CSK4/RAH or a PBS control. The results showed that Pam3CSK4 as an adjuvant could not enhance the anti-tumor response induced by the peptide vaccine (Figure 6). Thus, the triacylated lipopeptide (lipo-Nter) derived from a bacterial lipoprotein yields a better adjuvant effect in a peptide vaccine than the synthetic triacylated lipopeptide (Pam3CSK4).

## 4. Discussion

In this study, we purified lipo-Nter from the recombinant lipoprotein rlipo-D1E3 and analyzed its molecular weight using a MALDI micro MX mass spectrometer. Lipo-Nter is a triacylated peptide containing an unsaturated fatty acid in the R2 region and thus differs from the synthetic tripalmitoylated peptide (Pam3CSK4). The unsaturated fatty acid at R2 residue was elucidated by using collision induced dissociation of triacyl lipopeptide and its corresponding MS ions detected in MSn analysis [23]. To rule out the contamination of diacyl peptide in the purified lipo-Nter, mass spectrum of lipo-Nter was shown from* m/z* 1000 to 2500 in Figure S1(a) in Supplementary Material available online at http://dx.doi.org/10.1155/2014/349783. The purity of lipo-Nter was shown in Figure S1(b). Our data showed the coreceptor usage of the TLR2 ligation is dependent on TLR6 for lipo-Nter but is independent of TLR1 or TLR6 for Pam3CSK4. In addition, lipo-Nter can induce antitumor effects of a peptide vaccine, while Pam3CSK4 cannot. These results demonstrated that a lipopeptide derived from an* E. coli*-derived recombinant protein has potential for future application as a novel adjuvant for vaccine development.

Bacterial lipoproteins/peptides are major constituents of the cell wall of bacteria. These lipoproteins/peptides induce the innate immune response and promote the formation of adaptive immunity as an adjuvant during stimulation with specific antigens. The receptor responsible for a functional recognition of lipoproteins/peptides by cells is TLR2 [26, 27], which forms heterodimers with either TLR1 or TLR6 to attain specificity for a given stimulus [5, 12, 28]. However, the fatty acid composition of lipoprotein/peptide antigens affects their biological activity [29], and the molecular mechanism of the recognition of lipoproteins/peptides by the TLR2/TLR1 and TLR2/TLR6 heterodimers remains unclear. Many studies have demonstrated that diacylated or triacylated lipopeptides elicit humoral and cellular immune responses [30–35]. The responses obtained were generally comparable or superior to those obtained by immunization in combination with Freund's adjuvant. However, most of these studies used a synthetic lipopeptide containing a saturated fatty acid as a model to describe the adjuvant activity. Our results show that lipopeptides containing an unsaturated fatty acid have better antitumor effects than the synthetic lipopeptide Pam3CSK4.

An important finding of this work was that the TLR2 coreceptor usage of lipo-Nter was different from that of the parental lipoprotein rlipo-D1E3 or Pam3CSK4. The lipo-Nter activation of BM-DCs is dependent on TLR2/TLR6. We previously demonstrated that rlipo-D1E3 activated NF-*κ*B through the TLR2 signaling pathway and increased IL-23, IL-27, and MIP-1*α* expression by BM-DCs [15]. We also demonstrated that rlipoD1E3, like Pam3CSK4, activates BM-DCs independently of TLR1 or TLR6 [15]. We speculated that differences in the amino acid sequences or lengths of rlipo-D1E3 and lipo-Nter may play a role in their coreceptor usage. To confirm our hypothesis, a tripalmitoylated lipopeptide (Pam3CSQEAK) and a dipalmitoylated lipopeptide (Pam2CSQEAK) containing an amino acid sequence derived from lipo-Nter were synthesized (N-Palmitoyl-S-[2,3-bis(palmitoyloxy)-(2RS)-propyl]-[R]-cysteinyl-[S]-seryl-[S]-glutaminyl-[S]-glutamyl-[S]-alanyl-[S]-lysine or S-[2,3-bis(palmitoyloxy)-(2RS)-propyl]-[R]-cysteinyl-[S]-seryl-[S]-glutaminyl-[S]-glutamyl-[S]-alanyl-[S]-lysine), respectively. We determined that Pam3CSK4 and Pam3CSQEAK activated DCs using similar coreceptors (TLR1 or TLR6). Accordingly, Pam2CSK4 and Pam2CSQEAK activated DCs through TLR2/TLR6 (Figure S2). These data suggest that both the lipid moiety and amino acid component are important for coreceptor usage.

DCs play a key role in the stimulation of naive T cells and induce the differentiation of Th1 and Th2 cells [36, 37]. Th1 cells, which generate IFN-*γ* and IL-2, promote the cytotoxic functions of natural killer cells, CD8^+^ T cells, and macrophages. By contrast, Th2 cells, which induce IL-4, IL-5, and IL-10, promote humoral immunity mediated by B-cell-produced antibodies. We demonstrated that antigen immunization with lipo-Nter decreased the Th2 response and increased the Th1 response by attenuating IL-5 and inducing IL-2 (Figure 3). Unlike the recombinant lipoprotein rlipo-E7m [24], lipo-Nter did not greatly enhance IFN-*γ* secretion in response to rE7m immunization. However, the induction of IL-2 may play a critical role in CTL memory [38]. These results suggest that immunization with a TLR2 agonist-fused antigen may have different effects on the quality of CTL responses than those induced by immunization with a TLR2 agonist mixed antigen. Although the antitumor effects of lipo-Nter and rE7m were weaker than those of rlipo-E7m, the advantage of lipo-Nter is that it could be used for different types of antigens (i.e., synthetic peptides, viral particles, or cell lysates).

In summary, we have demonstrated that a lipid moiety containing an unsaturated fatty acid has been shown to activate APCs via the TLR2/TLR6 pathway and to induce Th1 phenotype development. Lipo-Nter could be used as an adjuvant in protein or peptide vaccines to induce specific CD8^+^ T cells and strong antitumor effects. The adjuvanticity of lipo-Nter for inducing an antitumor response is superior to that of synthetic Pam3CSK4. This lipid moiety may be developed as a novel adjuvant to stimulate immune responses.

## Highlights

- N-terminal fragment of lipoprotein rlipo-D1E3 (lipo-Nter) activated BMDCs via TLR2.
- Lipo-Nter elicits CTL and antitumor responses to protein and peptide vaccines.
- Lipo-Nter could be used as a novel adjuvant in a cervical cancer model.

## Supplementary Material

Supplementary Figure 1. One microliter of the polished tryptic fragments was mixed with 1*μ*l of a saturated solution of *α*-ciano-4-hydroxycinnamic acid in acetonitrile/0.1% trifluoroacetic acid (1:3, vol/vol). One microliter of the mixture was placed on the target plate of a MALDI micro MX mass spectrometer (Waters, Manchester, UK) for analysis. 3 *μ*g of purifed lipopeptides were loaded onto the Agilent 1100 series HPLC equipped with a 2.1x 100 mm POROS R1/10 column (Applied Biosystems®). The mobile phase A consisted of 0.1% formic acid in Milli-Q water and mobile phase B consisted of 0.1% formic acid in 100% CAN. a linear gradient was started from 5% to 100% B over a 20-min period and continued for 5 min at 100% B. After additional washing with 100% B for 5 min. Then, a steady gradient was washed with 20% B and 80% Isopropanol for 20 min. Later, we used 100% Isopropanol for additional washing, and change the buffer to 100% B. Finally, the buffer system was in 5% A and 95% B in 10 min.
Supplementary Figure 2. These lipopeptides were purchased from GeneDireX (Nevada, USA). These compounds are mixture of R and S stereoisomer. The stereochemistry of the compounds are: N-Palmitoyl-S-[2,3-bis(palmitoyloxy)-(2RS)-propyl]-[R]-cysteinyl-[S]-seryl-[S]-glutaminyl-[S]-glutamyl-[S]-alanyl-[S]-lysine (Pam3CSQEAK) and S-[2,3-bis(palmitoyloxy)-(2RS)-propyl]-[R]-cysteinyl-[S]-seryl-[S]-glutaminyl-[S]-glutamyl-[S]-alanyl-[S]-lysine (Pam2CSQEAK). BM-DCs were generated from wild-type (WT), TLR1^−/−^ (TLR1 KO), TLR2^−/−^ (TLR2 KO), or TLR6^−/−^ (TLR6 KO) mice as described in the manuscript. The cultured BM-DCs (1 x 10^6^/ml complete RPMI-10 medium) were stimulated with indicated lipopeptides (10 *μ*g/ml) or LPS (0.1 *μ*g/ml) for 24 h. The levels of TNF-*α* in the culture supernatant were measured by ELISA. LPS (TLR4 agonist), was used as positive control. LCM (complete RPMI-10 medium) was used as negative control. The results are expressed as the means + S.D. of the amount of cytokine.

## Abbreviations

TLR: Toll-like receptor
rE7m: Recombinant mutant E7 protein
Lipo-Nter: N-terminal of recombinant lipoprotein
Pam3CSK4: Synthetic lipopeptide (N-Palmitoyl-S-[2,3-bis(palmitoyloxy)-(2RS)-propyl]-CSKKKK).

## References

[1] Gearing AJ (2007). Targeting Toll-like receptors for drug development: a summary of commercial approaches. *Immunology & Cell Biology*.

[2] Iwasaki A, Medzhitov R (2004). Toll-like receptor control of the adaptive immune responses. *Nature Immunology*.

[3] Lahiri A, Das P, Chakravortty D (2008). Engagement of TLR signaling as adjuvant: towards smarter vaccine and beyond. *Vaccine*.

[4] West AP, Koblansky AA, Ghosh S (2006). Recognition and signaling by Toll-like receptors. *Annual Review of Cell and Developmental Biology*.

[5] Alexopoulou L, Thomas V, Schnare M (2002). Hyporesponsiveness to vaccination with *Borrelia burgdorferi* OspA in humans and in TLR1- and TLR2-deficient mice. *Nature Medicine*.

[6] Revets H, Pynaert G, Grooten J, De Baetselier P (2005). Lipoprotein I, a TLR2/4 ligand modulates Th2-driven allergic immune responses. *The Journal of Immunology*.

[7] Shimizu T, Kida Y, Kuwano K (2008). A triacylated lipoprotein from *Mycoplasma genitalium* activates NF-*κ*B through Toll-like receptor 1 (TLR1) and TLR2. *Infection and Immunity*.

[8] Tawaratsumida K, Furuyashiki M, Katsumoto M (2009). Characterization of N-terminal structure of TLR2-activating lipoprotein in *Staphylococcus aureus*. *The Journal of Biological Chemistry*.

[9] Thakran S, Li H, Lavine CL (2008). Identification of *Francisella tularensis* lipoproteins that stimulate the Toll-like receptor (TLR) 2/TLR1 heterodimer. *The Journal of Biological Chemistry*.

[10] BenMohamed L, Gras-Masse H, Tartar A (1997). Lipopeptide immunization without adjuvant induces potent and long-lasting B, T helper, and cytotoxic T lymphocyte responses against a malaria liver stage antigen in mice and chimpanzees. *European Journal of Immunology*.

[11] Buwitt-Beckmann U, Heine H, Wiesmuller K-H (2006). TLR1- and TLR6-independent recognition of bacterial lipopeptides. *The Journal of Biological Chemistry*.

[12] Takeuchi O, Sato S, Horiuchi T (2002). Cutting edge: role of Toll-like receptor 1 in mediating immune response to microbial lipoproteins. *The Journal of Immunology*.

[13] Akira S (2003). Mammalian Toll-like receptors. *Current Opinion in Immunology*.

[14] Farhat K, Riekenberg S, Heine H (2008). Heterodimerization of TLR2 with TLR1 or TLR6 expands the ligand spectrum but does not lead to differential signaling. *Journal of Leukocyte Biology*.

[15] Leng C-H, Chen H-W, Chang L-S (2010). A recombinant lipoprotein containing an unsaturated fatty acid activates NF-*κ*B through the TLR2 signaling pathway and induces a differential gene profile from a synthetic lipopeptide. *Molecular Immunology*.

[16] Kiura K, Kataoka H, Yasuda M, Inoue N, Shibata K-I (2006). The diacylated lipopeptide FSL-1 induces TLR2-mediated Th2 responses. *FEMS Immunology & Medical Microbiology*.

[17] Infante-Duarte C, Kamradt T (1997). Lipopeptides of *Borrelia burgdorferi* outer surface proteins induce Th1 phenotype development in *αβ* T-cell receptor transgenic mice. *Infection and Immunity*.

[18] Cottalorda A, Mercier BC, Mbitikon-Kobo FM (2009). TLR2 engagement on memory CD8^+^ T cells improves their cytokine-mediated proliferation and IFN-*γ* secretion in the absence of Ag. *European Journal of Immunology*.

[19] Cottalorda A, Verschelde C, Marcais A (2006). TLR2 engagement on CD8 T cells lowers the threshold for optimal antigen-induced T cell activation. *European Journal of Immunology*.

[20] Ghielmetti M, Zwicker M, Ghielmetti T, Simon MM, Villiger PM, Padovan E (2005). Synthetic bacterial lipopeptide analogs facilitate naive CD4^+^ T cell differentiation and enhance antigen-specific HLA-II-restricted responses. *European Journal of Immunology*.

[21] Prajeeth CK, Jirmo AC, Krishnaswamy JK (2010). The synthetic TLR2 agonist BPPcysMPEG leads to efficient cross-priming against co-administered and linked antigens. *European Journal of Immunology*.

[22] Chen H-W, Liu S-J, Liu H-H (2009). A novel technology for the production of a heterologous lipoprotein immunogen in high yield has implications for the field of vaccine design. *Vaccine*.

[23] Kwok Y, Sung W-C, Lin AL (2011). Rapid isolation and characterization of bacterial lipopeptides using liquid chromatography and mass spectrometry analysis. *Proteomics*.

[24] Huang CY, Chen JJ, Shen KY (2012). Recombinant lipidated HPV E7 induces a Th-1-biased immune response and protective immunity against cervical cancer in a mouse model. *PLoS ONE*.

[25] Mercier BC, Cottalorda A, Coupet C-A, Marvel J, Bonnefoy-Bérard N (2009). TLR2 engagement on CD8 T cells enables generation of functional memory cells in response to a suboptimal TCR signal. *The Journal of Immunology*.

[26] Lien E, Sellati TJ, Yoshimura A (1999). Toll-like receptor 2 functions as a pattern recognition receptor for diverse bacterial products. *The Journal of Biological Chemistry*.

[27] Takeuchi O, Kaufmann A, Grote K (2000). Cutting edge: preferentially the R-stereoisomer of the mycoplasmal lipopeptide macrophage-activating lipopeptide-2 activates immune cells through a Toll-like receptor 2- and MyD88-dependent signaling pathway. *The Journal of Immunology*.

[28] Morr M, Takeuchi O, Akira S, Simon MM, Muhlradt PF (2002). Differential recognition of structural details of bacterial lipopeptides by Toll-like receptors. *European Journal of Immunology*.

[29] Muller SD, Muller MR, Huber M (2004). Triacyl-lipopentapeptide adjuvants: TLR2-dependent activation of macrophages and modulation of receptor-mediated cell activation by altering acyl-moieties. *International Immunopharmacology*.

[30] Bessler WG, Baier W, vd Esche U (1997). Bacterial lipopeptides constitute efficient novel immunogens and adjuvants in parenteral and oral immunization. *Behring Institute Mitteilungen*.

[31] Bessler WG, Mittenbuhler K, Esche U, Huber M (2003). Lipopeptide adjuvants in combination treatment. *International Immunopharmacology*.

[32] Cataldi A, Yevsa T, Vilte DA (2008). Efficient immune responses against Intimin and EspB of enterohaemorragic *Escherichia coli* after intranasal vaccination using the TLR2/6 agonist MALP-2 as adjuvant. *Vaccine*.

[33] Deres K, Schild H, Wiesmuller K-H, Jung G, Rammensee H-G (1989). *In vivo* priming of virus-specific cytotoxic T lymphocytes with synthetic lipopeptide vaccine. *Nature*.

[34] Kerber-Momot T, Leemhuis D, Luhrmann A (2010). Beneficial effects of TLR-2/6 ligation in pulmonary bacterial infection and immunization with *Pseudomonas aeruginosa*. *Inflammation*.

[35] Schild H, Deres K, Wiesmuller K-H, Jung G, Rammensee H-G (1991). Efficiency of peptides and lipopeptides for *in vivo* priming of virus-specific cytotoxic T cells. *European Journal of Immunology*.

[36] Banchereau J, Steinman RM (1998). Dendritic cells and the control of immunity. *Nature*.

[37] Moser M, Murphy KM (2000). Dendritic cell regulation of TH1-TH2 development. *Nature Immunology*.

[38] Umeshappa CS, Xie Y, Xu S (2013). Th cells promote CTL survival and memory via acquired pMHC-I and endogenous IL-2 and CD40L signaling and by modulating apoptosis-controlling pathways. *PLoS ONE*.

