# Supplementary material for: A Purified Recombinant Lipopeptide as Adjuvant for Cancer Immunotherapy
Source: Biomed Res Int. 2014 Mar 11;2014:349783. doi: 10.1155/2014/349783 (PMC3967807; doi:10.1155/2014/349783)
Supplement: Supplementary file 1 — Supplementary Figure 1. One microliter of the polished tryptic fragments was mixed with 1μl of a saturated solution of α-ciano-4-hydroxycinnamic acid in acetonitrile/0.1% trifluoroacetic acid (1:3, vol/vol). One microliter of the mixture was placed on the target plate of a MALDI micro MX mass spectrometer (Waters, Manchester, UK) for analysis. 3 μg of purifed lipopeptides were loaded onto the Agilent 1100 series HPLC equipped with a 2.1x 100 mm POROS R1/10 column (Applied Biosystems®). The mobile phase A consisted of 0.1% formic acid in Milli-Q water and mobile phase B consisted of 0.1% formic acid in 100% CAN. a linear gradient was started from 5% to 100% B over a 20-min period and continued for 5 min at 100% B. After additional washing with 100% B for 5 min. Then, a steady gradient was washed with 20% B and 80% Isopropanol for 20 min. Later, we used 100% Isopropanol for additional washing, and change the buffer to 100% B. Finally, the buffer system was in 5% A and 95% B in 10 min. Supplementary Figure 2. These lipopeptides were purchased from GeneDireX (Nevada, USA). These compounds are mixture of R and S stereoisomer. The stereochemistry of the compounds are: N-Palmitoyl-S-[2,3-bis(palmitoyloxy)-(2RS)-propyl]-[R]-cysteinyl-[S]-seryl-[S]-glutaminyl-[S]-glutamyl-[S]-alanyl-[S]-lysine (Pam3CSQEAK) and S-[2,3-bis(palmitoyloxy)-(2RS)-propyl]-[R]-cysteinyl-[S]-seryl-[S]-glutaminyl-[S]-glutamyl-[S]-alanyl-[S]-lysine (Pam2CSQEAK). BM-DCs were generated from wild-type (WT), TLR1−/− (TLR1 KO), TLR2−/− (TLR2 KO), or TLR6−/− (TLR6 KO) mice as described in the manuscript. The cultured BM-DCs (1 x 10^6^/ml complete RPMI-10 medium) were stimulated with indicated lipopeptides (10 μg/ml) or LPS (0.1 μg/ml) for 24 h. The levels of TNF-α in the culture supernatant were measured by ELISA. LPS (TLR4 agonist), was used as positive control. LCM (complete RPMI-10 medium) was used as negative control. The results are expressed as the means + S.D. of the amount of cytokine. [file 349783.f1.pdf]

Figure S1

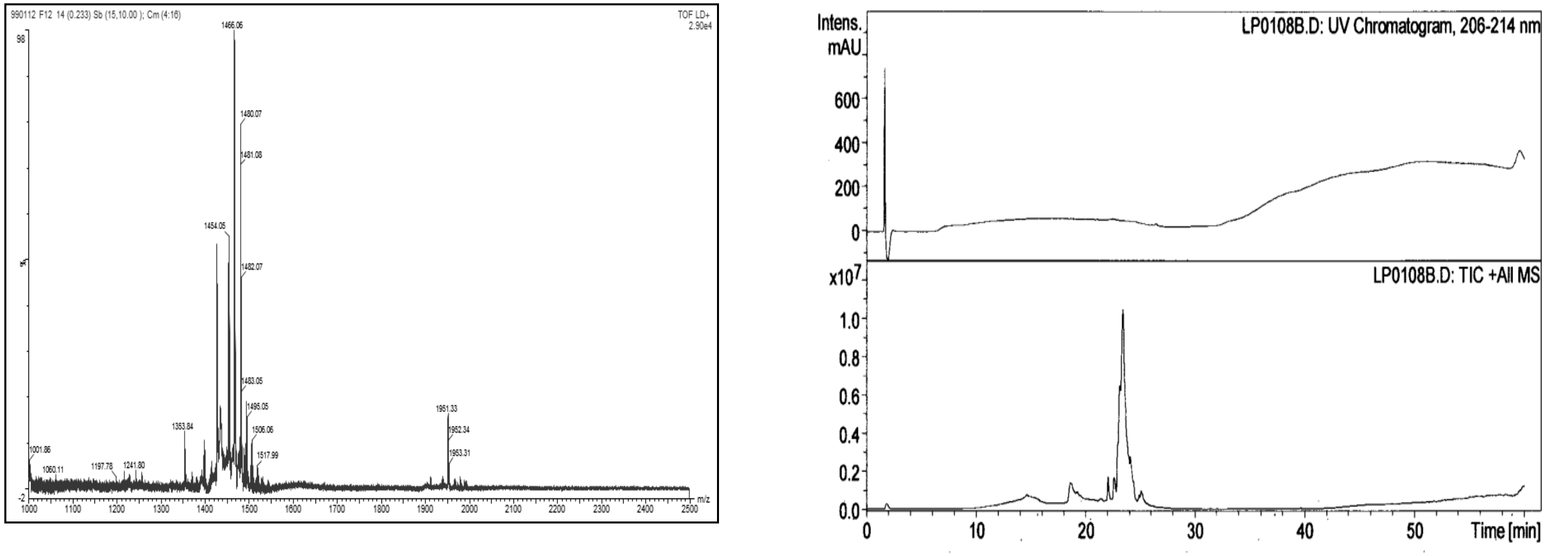

(a) (b)

Supplemental Figure 1. (a) The lipo-Nter was obtained after digesting the rlipo-D1E3 using trypsin and was analyzed on a Waters® MALDI micro MX™ mass spectrometer. The major peaks with m/z around1466 are the tri-acylpeptide. The sequences are the lipid moieties with CSQEAK. The peaks with m/z around 1951 are the tri-acylpeptide. The sequences are the lipid moieties with CSQEAKQEVK. The peaks of di-acylpeptides are in M/Z around 1228. No obvious peaks of di-acylpeptide were observed. (b) When checking the purity of lipo-Nter, no apparent peak was observed in the absorption spectrum at the range of 206-214 nm. Therefore, we showed the total ion current (TIC) with the absorption spectrum.

Figure S2

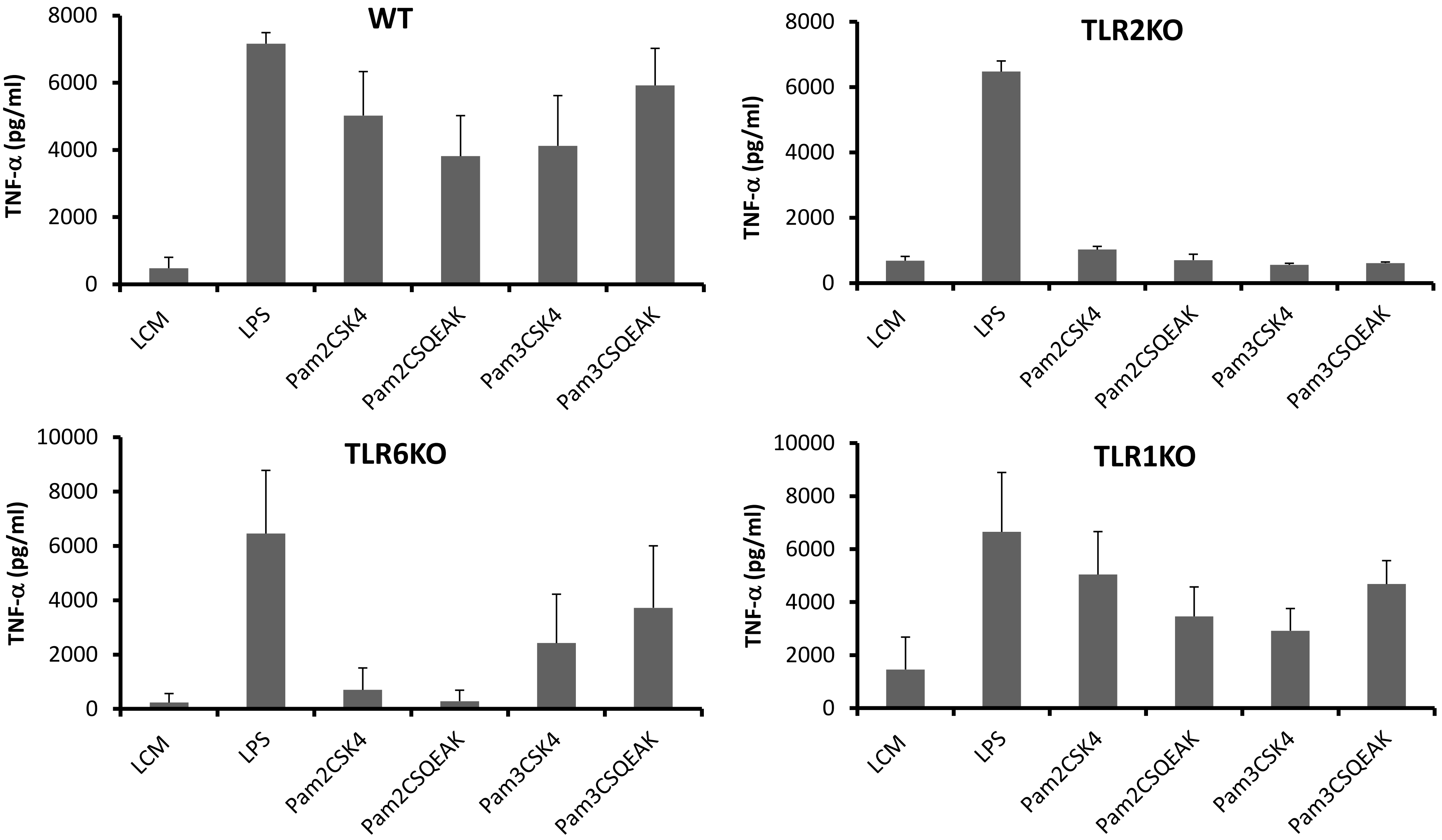

Supplemental Figure 2. The responses of BM-DCs after stimulation with lipidated peptide. BM-DCs were generated from wild-type (WT), TLR1$^{-/-}$ (TLR1 KO), TLR2$^{-/-}$ (TLR2 KO), or TLR6$^{-/-}$ (TLR6 KO) mice. BM-DCs were stimulated with indicated lipopeptide (10 μg/ml) or LPS (0.1 μg/ml) for 24 h. The levels of TNF-α in the culture supernatant were measured by ELISA. LPS (TLR4 agonist), was used as positive control. LCM (complete RPMI-10 medium) was used as negative control. The results are expressed as the means + S.D. of the amount of cytokine.

Figure S1

Supplemental Materials and Methods. One microliter of the polished tryptic fragments was mixed with 1μl of a saturated solution of α-ciano-4-hydroxycinnamic acid in acetonitrile/0.1% trifluoroacetic acid (1:3, vol/vol). One microliter of the mixture was placed on the target plate of a MALDI micro MX mass spectrometer (Waters, Manchester, UK) for analysis.

3 μg of purified lipopeptides were loaded onto the Agilent 1100 series HPLC equipped with a 2.1x 100 mm POROS R1/10 column (Applied Biosystems®). The mobile phase A consisted of 0.1% formic acid in Milli-Q water and mobile phase B consisted of 0.1% formic acid in 100% CAN. a linear gradient was started from 5% to 100% B over a 20-min period and continued for 5 min at 100% B. After additional washing with 100% B for 5 min. Then, a steady gradient was washed with 20% B and 80% Isopropanol for 20 min. Later, we used 100% Isopropanol for additional washing, and change the buffer to 100% B. Finally, the buffer system was in 5% A and 95% B in 10 min.

Figure S2

Supplemental Materials and Methods. These lipopeptides were purchased from GeneDireX (Nevada, USA). These compounds are mixture of R and S stereoisomer. The stereochemistry of the compounds are: N-Palmitoyl-S-[2,3-bis(palmitoyloxy)-(2RS)-propyl]-[R]-cysteinyl-[S]-seryl-[S]-glutaminyl-[S]-glutamyl-[S]-alanyl-[S]-lysine (Pam3CSQEAK) and S-[2,3-bis(palmitoyloxy)-(2RS)-propyl]-[R]-cysteinyl-[S]-seryl-[S]-glutaminyl-[S]-glutamyl-[S]-alanyl-[S]-lysine (Pam2CSQEAK).

BM-DCs were generated from wild-type (WT), $TLR1^{-/-}$ (TLR1 KO), $TLR2^{-/-}$ (TLR2 KO), or $TLR6^{-/-}$ (TLR6 KO) mice as described in the manuscript. The cultured BM-DCs (1 x 10^6/ml complete RPMI-10 medium) were stimulated with indicated lipopeptides (10 μg/ml) or LPS (0.1 μg/ml) for 24 h. The levels of TNF-α in the culture supernatant were measured by ELISA. LPS (TLR4 agonist), was used as positive control. LCM (complete RPMI-10 medium) was used as negative control. The results are expressed as the means + S.D. of the amount of cytokine.
